# Boolean Model of Yeast Apoptosis as a Tool to Study Yeast and Human Apoptotic Regulations

**DOI:** 10.3389/fphys.2012.00446

**Published:** 2012-12-10

**Authors:** Laleh Kazemzadeh, Marija Cvijovic, Dina Petranovic

**Affiliations:** ^1^Department of Chemical and Biological Engineering, Chalmers University of TechnologyGothenburg, Sweden; ^2^Digital Enterprise Research Institute, National University of IrelandGalway, Ireland; ^3^Department of Mathematical Sciences, Chalmers University of Technology and University of GothenburgGothenburg, Sweden

**Keywords:** apoptosis, Boolean modeling, Stm1, Bir1, Hog1, VCP, Bcl-2 family

## Abstract

Programmed cell death (PCD) is an essential cellular mechanism that is evolutionary conserved, mediated through various pathways and acts by integrating different stimuli. Many diseases such as neurodegenerative diseases and cancers are found to be caused by, or associated with, regulations in the cell death pathways. Yeast *Saccharomyces cerevisiae*, is a unicellular eukaryotic organism that shares with human cells components and pathways of the PCD and is therefore used as a model organism. Boolean modeling is becoming promising approach to capture qualitative behavior and describe essential properties of such complex networks. Here we present large literature-based and to our knowledge first Boolean model that combines pathways leading to apoptosis (a type of PCD) in yeast. Analysis of the yeast model confirmed experimental findings of anti-apoptotic role of Bir1p and pro-apoptotic role of Stm1p and revealed activation of the stress protein kinase Hog proposing the maximal level of activation upon heat stress. In addition we extended the yeast model and created an *in silico* humanized yeast in which human pro- and anti-apoptotic regulators Bcl-2 family and Valosin-contain protein (VCP) are included in the model. We showed that accumulation of Bax *in silico* humanized yeast shows apoptotic markers and that VCP is essential target of Akt Signaling. The presented Boolean model provides comprehensive description of yeast apoptosis network behavior. Extended model of humanized yeast gives new insights of how complex human disease like neurodegeneration can initially be tested.

## Introduction

Apoptosis is a complex process which is strictly under control of several regulatory networks. Any kind of malfunctioning in these controlling systems due to insufficient or excessive apoptosis signal can potentially lead to threatening diseases such as various types of cancer and neurodegenerative disorders. Therefore keeping this process tightly regulated is important for the cell. Even though apoptosis is often studied in multicellular organisms, the discovery of yeast apoptosis in 1997 (Madeo et al., 1997) attracted the attention of the wide research community (Frohlich et al., 2007; Owsianowski et al., 2008; Madeo et al., 2009; Carmona-Gutierrez et al., 2010b). As in other multicellular organisms the apoptosis in yeast is triggered by both internal and external signals. In yeast, the external signals can include acetic acid (Ludovico et al., 2001, 2002), salts, metal ions, ethanol, osmotic stress, heat stress (Madeo et al., 2009), lipids (Aerts et al., 2008; Low et al., 2008; Garbarino et al., 2009), mating pheromone (Zhang et al., 2006), different pharmacological molecules, and drugs (Almeida et al., 2008). Internal signals can include, ammonium, NO, ROS (that can be generated within the cell by mitochondria and the ER, and also induced by H_2_O_2_ addition (Madeo et al., 1999) and other factors), damage (proteins, lipids, nucleic acids) as a consequence of aging and mutations (Mazzoni et al., 2005; Weinberger et al., 2005; Hauptmann et al., 2006), as well as expression of heterologous proteins, such as human pro-apoptotic proteins (Eisenberg et al., 2007). Many proteins residing in the cytoplasm, nucleus, mitochondria, ER, peroxisomes, and lysosomes have been identified as the regulators of apoptosis. For example, proteolysis is one of the main steps that leads to execution of cell death and, a yeast metacaspase Yca1p has been shown to be central for most (but not all) cell death scenarios (Madeo et al., 2002, 2009). Besides degradation of proteins, degradation of nucleic acids is also carried out during apoptotic death and one of the two important caspase-independent mediators is Nuc1p (homolog of endonuclease G; Buttner et al., 2007). The second is Aif1p (apoptosis-inducing factor; Wissing et al., 2004) that is together with Nuc1p released from the mitochondrion and translocated to the nucleus during the initiation and execution of apoptosis. The regulation of apoptosis in the nucleus, is achieved via pro-apoptotic factor Nma111p (nuclear mediator of apoptosis; Fahrenkrog et al., 2004) a serine protease that cleaves an anti-apoptotic factor (inhibitor of apoptosis, IAP) Bir1p, which is the only known IAP in yeast, and its anti-apoptotic mechanisms (known to be YCA1-independent) are not well characterized (Walter et al., 2006). To understand how large and complex network of apoptosis process is regulated it should be studied as a whole allowing identification of the properties essential for biological function (Janes et al., 2005). To complement experimental studies mathematical models are often use permitting systematic analysis of the network components either individually or jointly (Wolkenhauer, 2002; Stelling, 2004). In Boolean networks (BN) introduced by Kauffman (1969) these assumptions are made based on activation/inhibition effects of one node on another downstream node. The Boolean “on” state (or 1 state or “true” state) can be translate to biological active state of specific species, while “off” state (or 0 state or “false” state) corresponds to inactive state. With simple logical rules (AND, OR, and NOT) it is possible to capture system’s behavior in a discrete manner without being dependent on experimental measurements such as molecular concentration or kinetic rates. This type of model implementation is becoming more common in biology (Handorf and Klipp, 2012) and examples include various model organisms and processes, ranging from cell cycle models of simple fission yeast (Davidich and Bornholdt, 2008), to complex dynamic analysis of mammalian cell cycle (Fauré et al., 2006), study of mammalian neurotransmitter signaling pathway (Gupta et al., 2007), investigation of irreversible mammalian apoptosis and stable surviving (Mai and Liu, 2009), and model of apoptosis in human (Schlatter et al., 2009). In each of these studies different extensions of Boolean modeling is implemented giving clear indication of the growing application of logic based modeling in qualitative studies of biological networks where there is not much quantitative data available.

We describe here a model based on Boolean network approach that consists of two parts: in the first part construction and the evaluation of the yeast apoptosis Boolean model is introduced and in the second part we propose the use of established model for study of human apoptotic proteins in yeast. Simplicity of BN allowed us to construct the model that integrates vast amount of heterogeneous knowledge that currently exists for yeast apoptosis. The major purpose of this study is to understand the emergence of systems properties. Extensive analysis of the state space in combination of different input signals generated series of *in silico* experiments. Simulating knock out experiments we were able to test the function of specific feedback structures in apoptotic network. The results were compared with the existing experimental data and the model was used to explore several hypotheses in order to better understand certain apoptotic mechanisms and to suggest new strategies for further experimental studies.

## Results

### Network topology

**Figure 1:**
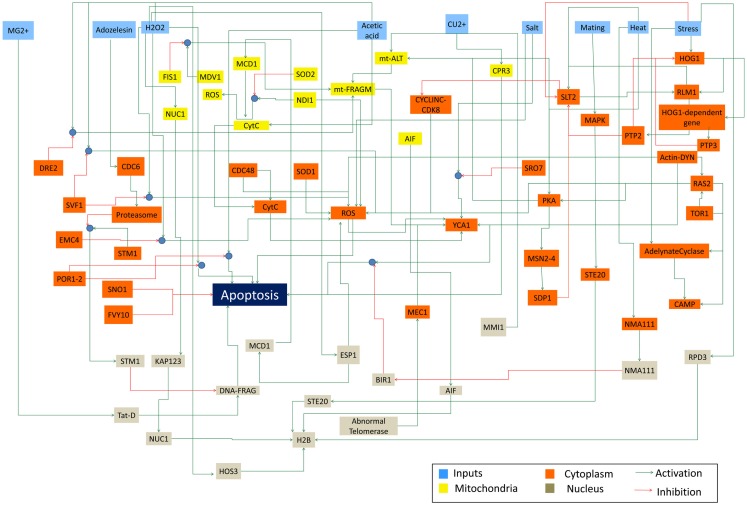
**Schematic representation of Yeast Apoptosis network**. Blue boxes depict input nodes, yellow nodes are placed in mitochondria, orange nodes reside in cytosol and grey nodes belong to nucleus. Green arrows show activation effect and red arrows show inhibition effect. Blue circles depict “AND” gate.

The constructed yeast apoptosis network contains 73 species and 115 reactions (Table 1). Six species do not have successor (Sink/Output) or predecessor (Source/Input; Table 2). In the schematic diagram (Figure 1) species are represented as nodes and reactions as edges. Species include: processes, proteins, and small molecules (metabolites or signals) and Reactions include *activation* (green arrow) and *inhibition* (red arrow). Nine nodes depict inputs to the model and are colored blue. Seventeen elements are active in nucleus and are shown in gray, 12 mitochondria species are shown in yellow, and 34 orange boxes represent species residing in cytoplasm. System has only one output node which is called “Apoptosis” and it is in dark blue. Filled blue circles are used as an “AND” gates between two or more reactions to indicate the necessity of presence of two species for activation or inhibition of a reaction (Figure 1).

**Table 1:** **List of species in yeast Boolean model**.

ID	Species	Type	Name description
1	ABNORMALTELOMERASE	Change	ABNORMALTELOMERASE
2	ACETIC ACID	Input	Chemical
3	ADENYLATECYCLASE	Enzyme	Lyase enzyme
4	ADOZELESIN	Input	Drug
5	AIF-MT	Protein	Apoptosis-inducing factor in mitochondria
6	AIF-NUC	Protein	Apoptosis-inducing factor in nucleus
7	APOPTOSIS	Output	Cell death
8	BIR1	Protein	Baculoviral IAP repeat-containing protein 1
9	CAMP	Protein	Cyclic adenosine monophosphate
10	CDC48	Protein	Cell division cycle
11	CDC6	Protein	Cell division cycle
12	CPR3	Protein	Cyclosporin-sensitive proline rotamase
13	CU2	Input	Ion
14	DesCYCLINCCDK8	Change	Destruction of cylinC/CDCk8
15	CYTC-CYT	Protein	CytochromC in cytososl
16	CYTC-MT	Protein	CytochromC in mitochondria
17	DNA-FRAG	Change	DNA fragmentation
18	DRE2/TAH18	Change	Dre2-TAH18 complex
19	EMC4	Protein	ER membrane protein complex
20	ESP1	Protein	Separase
21	FIS1	Protein	Mitochondrial FISsion
22	FYV10	Protein	Function required for yeast viability
23	H2B	Protein	Histon 2B
24	H2O2	Input	Hydroxide peroxide
25	HEAT	Input	Event
26	HK	–	House keeping function**
27	HOG1	Protein	High osmolarity glycerol response
28	HOG1-DEP	Protein	HOG1 dependent genes
29	HOS3	Protein	Hda one similar
30	KAP123	Protein	KAryoPherin
31	MAPK	Protein	Map kinase cascade
32	MATING	Input	Mating pheromone
33	MCD1-MT	Protein	Mitotic chromosome determinant in mitochondria
34	MCD1-NUC	Protein	Mitotic chromosome determinant in nucleus
35	MDV1	Protein	Mitochondrial DiVision
36	MEC1	Protein	Mitosis entry checkpoint
37	MG2	Input	Ion
38	MMI1	Protein	Translation machinery associated
39	MSN2-4	Protein	Multicopy suppressor of SNF1 mutation
40	MT-ALT	Change	Mitochondria alteration
41	MT-FRAG	Change	Mitochondria fragmentation
42	NDI1	Protein	NADH dehydrogenase internal
43	NMA111-CYT	Protein	Nuclear mediator of apoptosis in cytososl
44	NMA111-NUC	Protein	Nuclear mediator of apoptosis in nucleus
45	NUC1-MT	Protein	NUClease 1 in mitochondria
46	NUC1-NUC	Protein	NUClease 1 in nucleus
47	PKA	Protein	Protein kinase A
48	POR1-2	Protein	PORin
49	PROTOSOM	Complex	PROTOSOM
50	PTP2	Protein	Protein tyrosine phosphatase
51	PTP3	Protein	Protein tyrosine phosphatase
52	RAS2	Protein	Homologous to RAS proto-oncogene
53	RedActinDyn	Change	Reduced actin dynamic
54	RLM1	Protein	Resistance to lethality of MKK1P386 overexpression
55	ROS-CYT	Molecule	Reactive oxygen species in cytososl
56	ROS-MT	Molecule	Reactive oxygen species in mitochondria
57	RPD3	Protein	Reduced potassium dependency
58	SALT	Input	–
59	SDP1	Protein	Stress-inducible dual specificity phosphatase
60	SLT2	Protein	SYNtaxin (SYN8)
61	SNO1	Protein	SNZ proximal open reading frame
62	SOD1	Protein	Superoxide dismutase
63	SOD2	Protein	Superoxide dismutase
64	SRO7	Protein	Suppressor of rho3
65	STE20-CYT	Protein	Sterile in cytosol
66	STE20-NUC	Protein	Sterile in nucleus
67	STM1-CYT	Protein	Translation initiation factor (TIF3) in cytososl
68	STM1-NUC	Protein	Translation initiation factor (TIF3) in nucleus
69	STRESS	Input	Event
70	SVF1	Protein	SurVival factor
71	TAT-D	Protein	3′ → 5′ exonuclease and endonuclease
72	TOR1	Protein	Target of rapamycin
73	YCA1	Protein	MetaCAspase

*Table includes name, type, and description of each species involved in yeast apoptosis*.

*Species refers to all types of nodes that are depicted in the network map and are color coded based on their location or activity in cell.

**Housekeeping function refers to those genes which are present and give snapshot of state of the cell before applying any kind of treatment.

**Table 2:** **Summary of the interactions without successor (Sink/Output) and predecessor (Source/Input)**.

Species	Type of connection	Number of connections
Ros-MT	Sink/output	1
MCD1-NUC	Sink/output	1
H2B	Sink/output	5
CAMP	Sink/output	2
RedActinDyn	Source/input	3
AbnormalTelomer	Source/input	1
HK	Source/input	18

*Table contains species without successor (there are no edges coming out of these nodes) and predecessor (there are no edges going to these nodes)*.

Network is activated via input signals corresponding to the following species: Acetic Acid, Heat, H_2_O_2_, Adozelesin, Mg^2+^, Cu^2+^, Salt (NaCl), mating, and osmotic stress.

### Model properties

Like many molecular mechanisms in living cells, apoptosis can be approximated as an outcome of sequential regulation steps which do not occur all at the same time. As a result, upon induction, the state of the cell changes with passing time. In order to capture this feature, each reaction was assigned activation time scale implicating occurrences of different scenarios in sequential order. By introducing time delays to the logical function, it is possible to describe dynamic behavior of the given process using logical networks (Thomas and D’Ari, 1990).

For this study we used Cell Net Analyser (CNA; Klamt et al., 2006) which provides the function to capture signal propagation in a time series to get a snapshot of the network and discrimination of signaling events. This approach has been successfully used to model human apoptotic network revealing new feedback loops (Schlatter et al., 2009). To describe dynamic behavior of the yeast apoptosis five timescales *t* = [0, 2, 4, 5, 6] are assigned. These timescales are constants and indicate at which timescale each node gets activated or inhibited. Simulation of the network at *t* = *x* comprises of all interactions with timescale *t* ≤ *x* but not interactions with *t* ≥ *x*. This gives the possibility of separation of different functional groups such as different signaling pathways or feedback loops. It should be noted that specified timescales do not refer to real time but are only indicators of sequential regulatory steps (difference between timescale *t* = 0 and *t* = 2 is equal to difference between timescales *t* = 2 and *t* = 4 or *t* = 4 and *t* = 5 or between *t* = 5 and *t* = 6). Timescales are not assigned based on speed of reactions (how fast or slow reactions are), they are assigned based on the sequence of events. To allow flexibility in our model and facilitate changes and insertions of new events timescales 1 and 3 are reserved and not used in the current network structure. First timescale *t* = 0 is assigned to genes which are already present and constantly active in cell (27 reactions). Values for all input arcs to stimuli species are set to 1 at second timescale *t* = 2 (53 reactions). Further interactions are activated at time *t* = 4 (17 reactions). Although yeast apoptosis does not contain any feedback loops time point *t* = 5 is reserved for feedback loops which get activated in response to osmotic and heats shock and will lead to apoptosis. Considering impact of feedback loops on system behavior it is reasonable to assign them a separate timescale. Finally interaction occurring at the very end are assigned timescale *t* = 6 (18 reactions). Details on each reaction, their time points and species involved in reactions are in Table 3.

**Table 3:** **List of logical interactions in yeast Boolean model**.

ID	Interaction	Function	Time scale	Reference
1	HK = AIF1-MT	Housekeeping	0	
2	HK = DRE2/TAH18	Housekeeping	0	
3	HK = EMC4	Housekeeping	0	
4	HK = SVF1	Housekeeping	0	
5	HK = FVY10	Housekeeping	0	
6	HK = SOD2	Housekeeping	0	
7	HK = SNO1	Housekeeping	0	
8	HK = NDI1	Housekeeping	0	
9	HK = POR1-2	Housekeeping	0	
10	HK = MMI1	Housekeeping	0	
11	HK = MCD1-MT	Housekeeping	0	
12	HK = SRO7	Housekeeping	0	
13	HK = CDC48	Housekeeping	0	
14	HK = FIS1	Housekeeping	0	
15	HK = MDV1	Housekeeping	0	
16	HK = STM1-CYT	Housekeeping	0	
17	=AceticAcid	Input	2	
18	=Adozelesin	Input	2	
19	=CU2	Input	2	
20	=H_2_O_2_	Input	2	
21	=Mating	Input	2	
22	=MG2	Input	2	
23	=Salt	Input	2	
24	=Heat	Input	2	
25	=Stress	Input	2	
26	!SOD2 + NDI1 = ROS-MT		4	Li et al. (2006)
27	AceticAcid = CytC-MT		4	Ludovico et al. (2002)
28	CDC48 = CytC-CYT		4	Eisenberg et al. (2007)
29	CytC-CYT = YCA1		4	Eisenberg et al. (2007)
30	CytC-MT = CytC-CYT		4	Eisenberg et al. (2007)
31	MCD1-MT = CytC-MT		4	Yang et al. (2008)
32	MEC1 = YCA1		4	Weinberger et al. (2005)
33	MT-Frag = MT-ALT		4	Wissing et al. (2004)
34	!FYV10 = Apoptosis		4	Khoury et al. (2008)
35	2 CDC48 = ROS-CYT		4	–
36	CU2 + CPR3 = Apoptosis		4	Liang and Zhou (2007)
37	DNA-Frag = Apoptosis		4	Madeo et al. (2009)
38	ESP1 = ROS-CYT		4	Yang et al. (2008)
39	MT-Frag = YCA1		4	Eisenberg et al. (2007)
40	NMA111-CYT = NMA111-NUC		4	Walter et al. (2006)
41	NUC1-MT = KAP123		4	Buttner et al. (2007)
42	RAS2 = AdenylateCyclase		4	Wood et al. (1994)
43	RAS2 = ROS-CYT		4	Kataoka et al. (1984)
44	RedActinDyn = ROS-CYT		4	Eisenberg et al. (2007)
45	RedActinDyn = YCA1		4	Madeo et al. (2009)
46	ROS-CYT = Apoptosis		4	Eisenberg et al. (2007)
47	ROS-CYT = YCA1		4	Madeo et al. (2002)
48	Salt = ROS-CYT		4	Wadskog et al. (2004)
49	SOD1 = ROS-CYT		4	Eisenberg et al. (2007)
50	STE20-NUC = H2B		4	Madeo et al. (2009)
51	Stress = RPD3		4	Ahn et al. (2006)
52	Tat-D = DNA-Frag		4	Qiu et al. (2005)
53	!SNO1 = Apoptosis		4	Khoury et al. (2008)
54	AbnormalTelomer = MEC1		4	Weinberger et al. (2005)
55	AdenylateCyclase = CAMP		4	Schmelzle et al. (2004)
56	Adozelesin = CDC6		4	Blanchard et al. (2002)
57	AIF1-MT = AIF1-NUC		4	Wissing et al. (2004)
58	AIF1-NUC = H2B		4	Wissing et al. (2004)
59	Apoptosis =	Output	4	–
60	CDC6 = Protosom		4	Blanchard et al. (2002)
61	ESP1 = MCD1-NUC		4	Yang et al. (2008)
62	H_2_O_2_ = NUC1-MT		4	Buttner et al. (2007)
63	H_2_O_2_ = ESP1		4	Yang et al. (2008)
64	H_2_O_2_ = HOS3		4	Carmona-Gutierrez et al. (2010a)
65	Heat = NMA111-CYT		4	Walter et al. (2006)
66	HOS3 = H2B		4	Carmona-Gutierrez et al. (2010a)
67	KAP123 = NUC1-NUC		4	Buttner et al. (2007)
68	MAPK = STE20-CYT		4	Carmona-Gutierrez et al. (2010a)
69	MatingPheromone = MAPK		4	Carmona-Gutierrez et al. (2010a)
70	Mg^2+^ = Tat-D		4	Qiu et al. (2005)
71	MMI1 = MT-ALT		4	Eisenberg et al. (2007)
72	MT-ALT = MT-FRAG		4	–
73	NUC1-NUC = H2B		4	Buttner et al. (2007)
74	PKA = MT-ALT		4	Carmona-Gutierrez et al. (2010a)
75	RAS2 = MT-ALT		4	Eisenberg et al. (2007)
76	RAS2 = PKA		4	Carmona-Gutierrez et al. (2010a)
77	RedActinDyn = RAS2		4	Eisenberg et al. (2007)
78	RPD3 = H2B		4	Ahn et al. (2006)
79	STE20-CYT = STE20-NUC		4	Carmona-Gutierrez et al. (2010a)
80	Stress = AdenylateCyclase		4	Schmelzle et al. (2004)
81	TOR1 = CAMP		4	Schmelzle et al. (2004)
82	TOR1 = RAS2		4	Schmelzle et al. (2004)
83	Heat = SOD1		4	
84	2 NDI1 = ROS-CYT		4	–
85	Stress = TOR1		4	Schmelzle et al. (2004)
86	!PTP2 = SLT2		5	Hahn and Thiele (2002)
87	!PTP2 = HOG1		5	Hahn and Thiele (2002)
88	HOG1 = HOG1-Dep		5	Hahn and Thiele (2002)
89	!PTP3 = HOG1		5	Hahn and Thiele (2002)
90	!SDP1 = SLT2		5	Hahn and Thiele (2002)
91	!SLT2 = DesCyclinCCDK8		5	Krasley et al. (2006)
92	!Stress = SLT2		5	Hahn and Thiele (2002)
93	DesCyclinCCCDK8 = ROS-CYT		5	Krasley et al. (2006)
94	Heat = PKA		5	Hahn and Thiele (2002)
95	Heat = SLT2		5	Hahn and Thiele (2002)
96	Hog1 = RLM1		5	Hahn and Thiele (2002)
97	HOG1-Dep = PTP3		5	Hahn and Thiele (2002)
98	MSN2-4 = SDP1		5	Hahn and Thiele (2002)
99	PKA = MSN2-4		5	Hahn and Thiele (2002)
100	RLM1 = PTP2		5	Hahn and Thiele (2002)
101	RLM1 = SLT2		5	Hahn and Thiele (2002)
102	SLT2 = RLM1		5	Hahn and Thiele (2002)
103	Stress = HOG1		5	Hahn and Thiele (2002)
104	!SRO7 + Salt = YCA1		6	Wadskog et al. (2004)
105	!STM1-NUC = DNA-Frag		6	Ligr et al. (2001)
106	AceticAcid + !SVF1 = ROS-CYT		6	Vander Heiden et al. (2002)
107	H_2_O_2_ + !EMC4 = ROS-CYT		6	Ring et al. (2008)
108	H_2_O_2_ + !SVF1 = ROS-CYT		6	Vander Heiden et al. (2002)
109	!FIS1 + MDV1 = MT-Frag		6	Eisenberg et al. (2007)
110	!POR1-2 + AceticAcid = Apoptosis		6	Pereira et al. (2007)
111	!POR1-2 + H_2_O_2_ = Apoptosis		6	Pereira et al. (2007)
112	H_2_O_2_ + !DRE2/TAH18 = MT-Frag		6	Vernis et al. (2009)
113	YCA1 + !BIR1 = Apoptosis		6	Walter et al. (2006)
114	!NMA111-NUC = BIR1		6	Walter et al. (2006)
115	STM1-CYT + !Protosom = STM1-NUC		6	Ligr et al. (2001)

*Table includes involved species in each interaction, logical rule for each interaction and time scale in which interaction takes place. Note: the interactions of the model are given in the notation of the Cell Net Analyser: a logical NOT is represented by “!”; a logical AND is represented by “+” and interaction on right hand side of “=” gives the value of node on left hand side or equation*.

The model simulated the induction of apoptosis both in the independent mode (assessing each individual stimulus separately) as well as in the additive mode, where the activation of all inputs were set at the same time (Table 4).

**Table 4:** **Predicted states of relevant species at steady state**.

Species	*t* = 0	*t* = 4	*t* = 5	*t* = 6
Acetic acid	1	1	1	1
Adozelesin	1	1	1	1
AIF1-MT	1	1	1	1
AIF1-NUC	0	1	1	1
Apoptosis	0	0	0	1
BIR1	0	0	0	0
DNA-FRAG	0	1	1	1
H2O2	1	1	1	1
Heat	1	1	1	1
NMA111-NUC	0	1	1	1
ROS-CYT	0	1	1	1
STE20-CYT	0	1	1	1
STM1-CYT	1	1	1	0
STM1-NUC	0	0	0	1
YCA1	0	1	1	1

*Table includes time scale scenario upon induction of all stimuli. T stands for different time steps. As an example if Acetic Acid is inducted to the cell at time point 0 it is expected to cause DNA Fragmentation at next time step and eventually ends to apoptosis in last step*.

### Predictions with the continuous model

Based on qualitative knowledge we have constructed a discrete Boolean model of yeast apoptosis. While it can capture its essential behavior, the question remained how this model can be used to predict the qualitative behavior of the system. In order to address this question we expanded the model by transforming the discrete Boolean model into a continuous model using Odefy (Wittmann et al., 2009; Krumsiek et al., 2010; see Materials and Methods). In order to test the continuous model and its predictive capacity, we performed three independent case-studies: (i) induction of apoptosis by activation of Hog1p by heat stress, (ii) inhibition by Bir1p of acetic acid-induced apoptosis, and (iii) induction of apoptosis with H_2_O_2_ by activation of Stm1p. We show here that the behavior that emerges from specific interactions in the model is in agreement with published experimental data.

### Activation of heat stress-induced apoptosis with Hog1p

**Figure 2:**
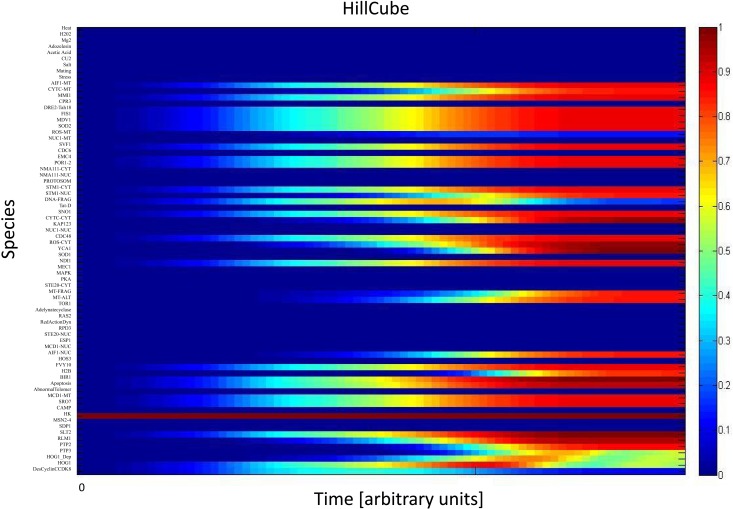
**Continuous Hill Cube transformation**. All species in apoptosis network are mapped on vertical axis. Dark blue color indicates those nodes that are not activated (value = 0) while dark red refers to nodes which are completely activated (value = 1). Each color in between indicates the level of activation between 0 and 1.

**Figure 3:**
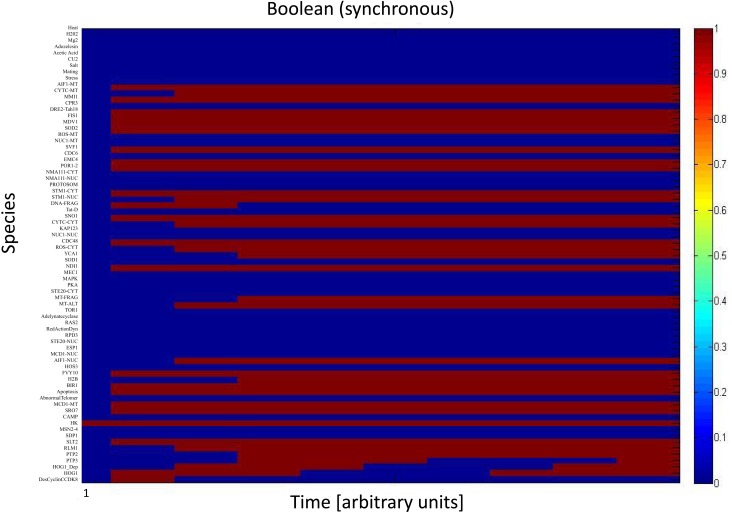
**Synchronous Boolean model**. All species in apoptosis network are mapped on vertical axis. Dark blue indicates those nodes that are not activated (value = 0) while dark red refers to nodes which are totally activated (value = 1).

**Figure 4:**
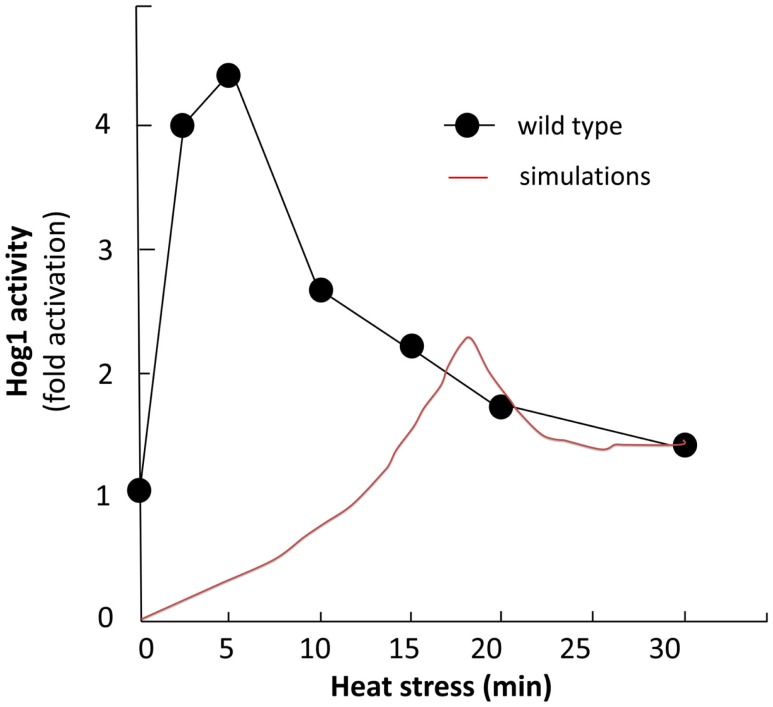
**Hog1 study**. Comparison of experimental and simulation study. Black curve shows activity of wild type Hog1 upon induction of heat (Winkler et al., 2002) and red curve illustrates Hog1 activation level in continues simulation.

We simulated the activation of the mitogen-activated protein kinase that is a key component of the HOG pathway (Albertyn and Hohmann, 1994; Van Wuytswinkel et al., 2000; Hohmann, 2002; de Nadal et al., 2004), which is an osmoregulatory signal transduction cascade (Hohmann, 2009). Upon stress, Hog1p (encoded by HOG1/YLR113W) regulates the expression of almost 600 genes by phosphorylating several different transcription factors (Hohmann, 2002; Westfall et al., 2004). The activity and nuclear localization of Hog1p is regulated by its phosphorylation state, and that in turn is regulated by the kinase MAPKK Pbs2p and the phosphatases Ptc1p, Ptc2p, Ptc3p, Ptp2p, and Ptp3p (Brewster et al., 1993; Ferrigno et al., 1998; Warmka et al., 2001; Young et al., 2002). Besides the induction by osmotic stress, the HOG pathway can be induced by heat stress, *via* a Sho1p-dependent sensory mechanism (Winkler et al., 2002), thus we used heat stress as an input to activate the HOG pathway and simulate the cell death response. Heat stress activates Slt2 which then activates Rlm1. Consequently Rlm1 triggers expression of Slt2 and PTP2 forming a feedback loop. On the other hand heat shock inhibits activity of PKA leading to release the inhibition effect of PKA on MSN2-4. MSN2-4 activates Sdp1 which then along with PTP2 inhibit Slt2. We started by setting all other stimuli to zero and then by transforming the Boolean apoptosis model into Hill Cube continuous model we performed the simulations. Steady states predicted from continuous Hill Cube (Figure 2) and synchronous Boolean (Figure 3) model are in perfect agreement with each other for all nodes apart from the nodes corresponding to the feedback loops in response to heat and osmotic stress. Feedback loop in heat activated pathway includes activation of Rlm1 by Slt2 and expression of Slt2 by Rlm1. Osmotic shock induced Hog1 activates Rlm1 to regulate Slt2 which was inhibited by osmotic shock and PTP2. Unknown Hog1 dependent transcription factor triggers transcription of PTP3 and PTP2 resulting in dephosphorylation of Hog1 phosphotyrosine which inhibits Hog1 activities. All nodes in our model were connected to activation of apoptosis *via* the heat shock pathway and were indeed activated in our simulation and are colored in red; other pathways are colored in blue indicating that they are not activated during the simulation (Figure 2). Model predicts that upon heat induction, concentration of Hog1p changes trough time never reaching its maximum level of concentration and as an intensity of stimulus decreases level of Hog1p also decreases. Our simulations also proposed that the maximal level of activation of Hog1p during heat stress is 70% of the total activation and as the heat stimulus continues over time its activity decreases and reaches a plateau at 40% of total activation (Figure 4).

This model prediction is in agreement with an experimental measurement performed by Winkler et al. (2002) which shows the induced activity of Hog1p upon heat induction (Figure 4). The difference between the maximal activity values and the lag phase of the activation is due to the fact that we did not use any quantitative data as input to the model.

### Inhibition of acetic acid-induced apoptosis with Bir1p

Even though there are several genes and pathways that are involved in yeast pro-apoptotic response, there are only few anti-apoptotic genes. For many years Bir1p (encoded by BIR1/YJR089W) was thought to be the only apoptotic inhibitor in yeast, but recently a TSC22-protein family was found and two of the proteins with TSC22-motif have been shown to have anti-apoptotic roles Sno1p and Fvy10p (Khoury et al., 2008). Bir1 belongs to the IAP family and phylogenetic analysis (Uren et al., 1998) showed similarity to *Schizosaccharomyces pombe* BIR1, human survivin, and *Caenorhabditis elegance* BIR-1 and BIR-2 proteins. Yeast’s Bir1p has been previously intensively studied in chromosome segregation (as a component of the Aurora kinase complex (chromosome passenger complex, CPC; Ruchaud et al., 2007) but recently studies of its role as a negative regulator of apoptosis have gained momentum. It has been shown that Bir1p is a target for degradation by Nma111p (Owsianowski et al., 2008), an apoptotic serine protease and yeast cells lacking Bir1 are more sensitive to apoptosis, while overexpression of Bir1 reduces apoptosis (Walter et al., 2006).

**Figure 5:**
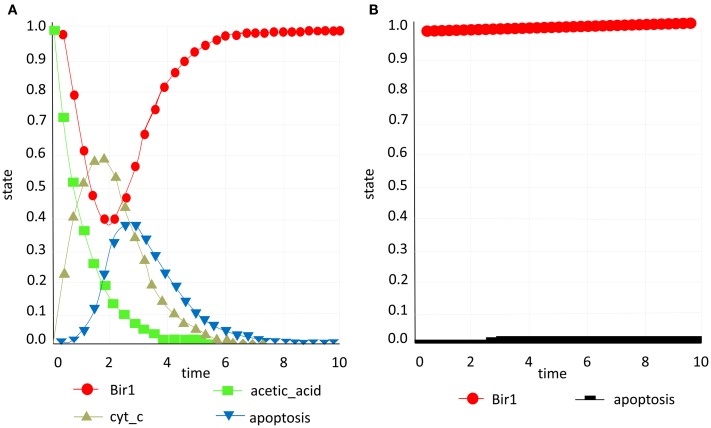
**Bir1 study**. **(A)** Acetic acid (green) is applied as a pulse stimulus (that is then decreasing over time) and cytochrome *c* (yellow) present in the cytosol increases, the decrease of Bir1p (red) promotes apoptosis **(B)** Constant presence of Bir1p (red) inhibits apoptosis (black), validating Bir1 anti-apoptotic role.

When building the model we assumed that addition of acetic acid induces cytochrome *c* release from mitochondria and its translocation to the cytosol. Another assumption is that the execution of apoptosis is Yca1p-dependent (encoded by YCA1/MCA1/YOR197W) in this context and that the activation of the caspase is downstream from the cytochrome *c* translocation (Ludovico et al., 2001, 2002). Thus, taking these assumptions into account acetic acid was used as an inducer in the simulations with direct induction of apoptosis. The model confirmed experimental evidence that Bir1 is indeed apoptosis inhibitor in yeast (Walter et al., 2006) and as acetic acid is applied as a pulse stimulus (that is then decreasing over time) and cytochrome *c* present in the cytosol increases, the decrease of Bir1p due to Nma111p degradation promotes apoptosis that then reaches the maximum (Figure 5A). We then performed an artificial and biologically irrelevant simulation: after apoptosis has occurred and no more inducer was present (acetic acid is not added again and the previously added amount has been “used”) the eventual theoretical accumulation of Bir1p did inhibit apoptosis and revert it to zero. Obviously this scenario is biologically implausible, since once the cell has undergone apoptosis it cannot be revived, but has a purpose to show mathematical correctness of the developed model.

Additionally, we have converted discreet apoptosis model to continuous model with constant presence of Bir1 in order to test if this type of model would predict the same outcome as the experimental approach in which Bir1p is overexpressed and provides protection from induction of apoptosis. The continuous model was created taking into account translocation effect of each element from one compartment to another. This effect has been applied to translocation of Nuc1 from mitochondria to nucleus, MCD1 from nucleus to mitochondria, Stm1 from cytosol to nucleus Aif1 (from mitochondria to nucleus, NMA111 from cytosol to nucleus, Ste20 from cytosol to nucleus, and cytochrome *c* from mitochondria to cytosol. Initially, in this model, translocation of each species is modeled as one node in order to represent the re-localization which introduced self-regulatory loops to the system which is impossible since to our knowledge yeast apoptosis regulatory network does not have such loops. One solution implies intuitive approach in defining single variable for single species to mimic the effect of transferring from one compartment to another. Technically this was solved by introducing two variables for a single species representing both compartments they can belong to. This approach, also, confirmed experimental finding (Walter et al., 2006) that constant presence of Bir1, inhibits apoptosis, validating Bir1 anti-apoptotic role (Figure 5B).

### Activation of H_2_O_2_-induced apoptosis with Stm1p

It has been shown that degradation of short-lived pro-apoptotic proteins via proteasomal – ubiquitine pathway plays important role in mammalian apoptosis (Drexler, 1997). One of the yeast proteasomal substrates – Stm1 (YLR150W) was identified in the study by Ligr et al. (2001) as an activator of the cell death process. Conserved orthologs of Stm1 are detected in several species (highly conserved ortholog in *Schizosaccharomyces pombe* and a putative ortholog in *Drosophila melanogaster* (Nelson et al., 2000) suggesting that regulation of apoptosis via this protein can be evolutionary conserved process.

**Figure 6:**
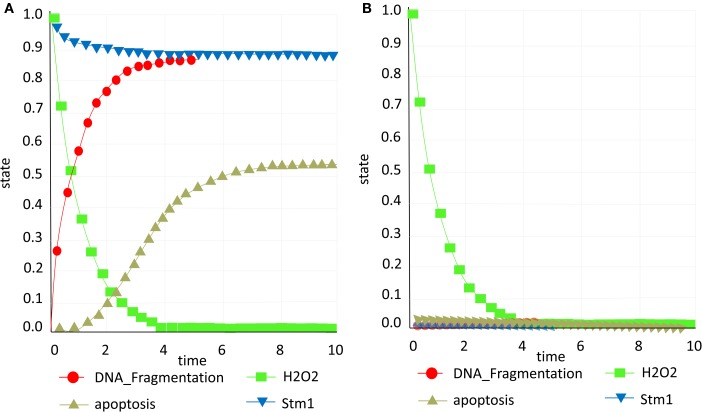
**Stm1 study**. **(A)** Presence of Stm1p (blue) promotes apoptosis (yellow; **B)** Knock out of Stm1p (blue) prevents apoptosis (yellow) and DNA fragmentation (red) and consequently promotes survival.

We have *in silico* tested two scenarios confirming the role of Stm1 in apoptosis (Ligr et al., 2001). Accumulation of Stm1 will induce apoptotic behavior followed by DNA fragmentation which is a known marker of cell death, while the Stm1 knock out will promote survival and consequently deterring DNA fragmentation (Figures 6A,B).

### *In silico* humanized yeast apoptosis

**Figure 7:**
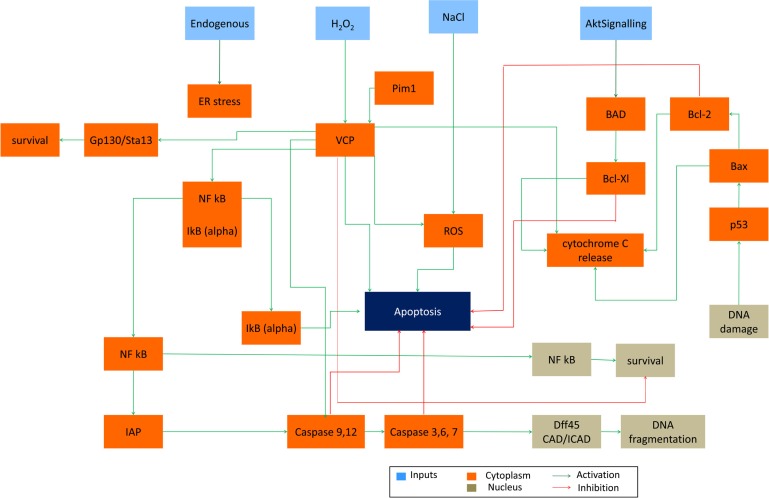
***In silico* “humanized yeast apoptosis network”**. Human apoptotic pathways BCL-2 protein family and VCP dependent genes inserted into the yeast apoptosis network (due to simplicity we show only human pathways).

*S. cerevisiae* is a model organism that has conserved genes, proteins and pathways that are similar to the ones in the human cells. This allows for using yeast as a host in which human genes can be expressed, so called “humanized yeast” (Winderickx et al., 2008) and subsequently the physiological roles and molecular mechanisms can be studied. In order to test if our system could be used in a similar way (*in silico* humanized yeast) and provide reliable simulations and predictions, we inserted three human genes with complete downstream pathways into our initial yeast apoptosis Boolean model. Yeast apoptotic network was “humanized” by *in silico* insertion of genes belonging to Bcl-2 protein family and Valosin-contain protein (VCP) – distinctive representatives of human apoptosis (Figure 7).

### Insertion of Bcl-2 family proteins: Anti-apoptotic Bcl-xL and pro-apototic bax

This protein family consists of apoptotic agonist and anti-agonist proteins. Among members of Bcl-2 family Bax as an apoptotic inducer and Bcl-xL as an anti-apoptotic factor were included in our extended model. Bax is located in cytosol in mammalian cells and has vital role in mitochondria morphogenesis. Heterologously expressed Bax causes growth arrest and rapid cell death in *S. cerevisiae* (Sato et al., 1994; Greenhalf et al., 1996). Also expression of Bax has been linked to the release of cytochrome *c* from mitochondria (Manon et al., 1997). Yeast cells with Bax expression accumulate ROS and show other apoptotic hallmarks such as DNA fragmentation (Madeo et al., 1999).

Since members of this protein family were previously successfully expressed in yeast, we first validate the “humanized” yeast model by inserting Bcl-xL, Bax, Bad, and Bcl-2 genes (Sato et al., 1994; Greenhalf et al., 1996; Madeo et al., 1999). As an input signal human Akt signaling was used (Akt Signaling), as an extrinsic regulatory switch (since this signaling cascade is not present in yeast). The Akt Serine/Threonine-kinase promotes cell survival by phosphorylating the pro-apoptotic protein BAD (member of the Bcl-2 family), which is the cause of dissociation of BAD from the Bcl-2/Bcl-X complex, and promotion of cell survival (Datta et al., 1997). Besides cell survival, Akt signaling is related to the cell cycle, metabolism, and angiogenesis and therefore a target for anticancer drug development (Falasca, 2010; Hers et al., 2011).

Upon activation of Akt Signaling at the first time step (*t* = 0, node value Akt Signaling = 1), Bcl-2 and Bcl-xL are activated in the following step (*t* = 4, node value Bcl-xL = 1 and Bcl-2 = 1) and remain active throughout the simulation and as the inhibitors of apoptosis promote the survival (node value Apoptosis = 0; Table 5). Simulation results suggest the anti-apoptotic role of two Bcl-2 family members: Bcl-2 and Bcl-xL which is consistent with experimental findings (Kharbanda et al., 1997).

**Table 5:** **Summary of simulations upon insertion of Bcl-2 pathway**.

Species	*t* = 0	*t* = 4	*t* = 5	*t* = 6
UV	0	0	0	0
Akt signaling	1	1	1	1
BCL-XL	0	1	1	1
P53	0	0	0	0
BAD	0	0	0	0
BAX	0	0	0	0
BCL-2	0	1	1	1
Apoptosis	0	0	0	0

*Table includes simulation results of heterologous expression of BCL-2 in yeast apoptosis model. T stands for different time steps. Upon activation of Akt Signaling pathway at first time step BCL-2 gets activated in following step and as an inhibitor of apoptosis prevents apoptosis till end point*.

### Dual functionality of VCP in survival and apoptosis

The evolutionary conserved Valosin-containing protein (VCP) is a mammalian ortholog of yeast Cdc48, which is the first apoptotic mediator found in *S. cereivisae* (Braun and Zischka, 2008). VCP is the member AAA-ATPase family which is ubiquitously expressed (Braun and Zischka, 2008). It consists of four domains: N-terminal domain, two ATPase domains D1 and D2 and C-terminal domain (Wang et al., 2004). The major ATPase activity of VCP is carried on by D2 domain (Wang et al., 2004; Song et al., 2003). VCP is involved in different cellular process such as protein degradation, membrane fusion and chaperone activity (Braun and Zischka, 2008). Role of VCP/Cdc48 in fluctuating number of death cell in various types of disease naming cancer and protein deposition diseases is not well understood. Increase in expression of VCP is correlated to the development of cancer and metastasis therefore detecting the level of VCP expression is proposed as cancer progression marker. Also VCP is known as detector of aggregated proteins causing neurodegenerative disease such as Parkinson and Alzheimer (Hirabayashi et al., 2001; Mizuno et al., 2003; Ishigaki et al., 2004). It has been observed that yeast cell carrying mutation of Cdc48 is showing morphological markers of apoptosis (Madeo et al., 1997) and that depletion of this protein has apoptotic effect in other organisms as well (Imamuraab et al., 2003).

It had been shown that VCP can both promote or inhibit apoptosis (Braun and Zischka, 2008). Deletion of VCP triggers ER stress which is followed by unfolded protein response and cell death via ER-associated degradation (ERAD) pathway – a highly conserved pathway between mammalian and yeast cells (Braun and Zischka, 2008). Wild type VCP has pro-apoptotic role in the cells undergoing apoptosis upon ER stress which in turn triggers ER-associated apoptotic pathway. Finally VCP can trigger survival pathway in response to NFkB which is a pro-survival molecule in cells with over expressed level of VCP/Cdc48(Braun and Zischka, 2008).

The dual functionality of VCP in survival and apoptosis mechanism makes it an attractive candidate to be inserted in initial yeast apoptosis Boolean network. Since VCP includes expression of a handful of heterologous proteins, its *in vivo* insertion to yeast plasmid would be difficult to perform and thus represents a good candidate for further investigation and exploration of model capabilities.

In our model, yeast Cdc48 was replaced with human VCP gene and its downstream pathway effector caspases (caspases 3, 6 and 7), initiator caspases (caspase9 and 12), IAP family, IkB-alpha inhibitory protein which when degraded by proteasome cause the release, and nuclear translocation of active NFkB (represented in the model as NFkB_Cyt and NFkB_Nuc) and gp130/Stat3 pathway (represented as a single node).

Upon activation of Akt Signaling pro-survival role of VCP is observed (node Survival = 1 at *t* = 6, Table 3). This is achieved when IkBα is dissociated from NFkB (node NFkB/IkBα = 1 at *t* = 4), which in turn gets activated (node NFkB = 1, *t* = 5) and is translocated to the nucleus (nodes NFkB_Cyt = 1 and NFkB_Nuc = 1, *t* = 6) inducing survival of the cell (node Survival = 1, *t* = 6). Simultaneously, activation of NFkB, promotes activity of IAP family of proteins which inhibits the activity of both effector and initiator caspases (nodes c-3-6-7 = 0 and c-9-12 = 0, *t* = [0,4,5,6], thus disabling apoptosis-dependent caspase pathway. As an independent, but parallel process, activation of VCP via Akt Signaling activates gp130-Stat3 (node gp130-Stat3 = 1, *t* = 4) pathway and consequently leads to survival (Table 6).

**Table 6:** **Summary of simulations upon insertion of VCP pathway**.

Species	*t* = 0	*t* = 4	*t* = 5	*t* = 6
Akt signaling	1	1	1	1
GP130-STAT3	0	1	1	1
NFkB-CYT	0	1	1	1
NFkB-NUC	0	1	1	1
NFkB/IkBα	0	1	1	1
VCP	1	1	1	1
C-3-6-7	0	0	0	0
C-9-12	0	0	0	0
IkBα	0	1	1	1
IAP	0	1	1	1
Survival	0	0	0	1
Apoptosis	0	0	0	0

*Table includes simulation results of humanized yeast apoptosis model by insertion of VCP. T stands for different time steps. VCP in presence of Akt Signaling is expected to prevent apoptosis and promote survival at last time point*.

This result suggests that yeast carrying mammalian VCP gene exhibits the same behavior as it is known from the human apoptotic model (Vandermoere et al., 2006).

## Discussion

In this work we have constructed a Boolean model for the biochemical network that controls apoptosis pathway in budding yeast *Saccharomyces cerevisiae*. Firstly, we presented the Boolean model describing only yeast pro and anti-apoptotic genes and validated the model by further analyzing the role of Stm1p, Bir1p, and Hog1p. Even though construction of the yeast apoptosis network involved certain simplifications (nodes have only two states and rules are describing network dynamics) we were able to model more complex network than using dynamic modeling approach that requires knowledge of kinetic parameters for all molecular processes. This approach was able to suggest general design principals of yeast apoptosis. One of the advantages of constructing mathematical models in biology is that we are able to simulate scenarios that are not feasible in real experiments. In the case of Bir1 *p* we were able to *in silico* revive a cell already reaching apoptosis. These examples are important to understand general mechanisms of the constructed network. A study of proteasomal substrate Stm1p suggested that Stm1p seems to be an effector of H_2_O_2_ and the presence of Stm1p with an inducer leads to apoptosis in our model, and in Stm1p knock out survival is promoted and DNA fragmentation is avoided, irrespective of the presence of the inducer. Despite the limitation of BN in terms of giving quantitative predictions of the system dynamics they allow investigation of the large networks and their systematic exploration resulting in better understanding of cellular processes. Example of Hog1p study showed that our model was capable of reproducing the pattern of Hog1p activation, but was not able to quantitatively predict the maximal Hog1p activity.

In the second stage, we extended the initial model by inserting two pathways of human apoptosis (VCP and Bcl-2 family), hereby creating a “humanized” *in silico* yeast. Humanized yeast strains are used in experimental molecular biology where the human proteins (causing, or associated with diseases) are expressed and studied *in vivo* (in yeast in this case).

Conservation of many apoptotic mediators and mechanisms among the *Eukarya* provides the possibility of inserting genes from other organisms into yeast (*in vivo*) or in the yeast apoptotic network (*in silico*). We validated constructed humanized yeast model by insertion of Bcl-2 family which have been previously successfully expressed in yeast. The model was able to reproduce well-known pro and anti-apoptotic phenotypes confirming that yeast expressing Bax accumulated ROS and showed other apoptotic markers like DNA fragmentation. To test whether or not specific pathways playing role in neurodegeneration and cancer can be elucidated by our Boolean model and can we consequently take advantage of a simple system like yeast to explore hypothesis generated for higher organisms, we *in silico* expressed evolutionary conserved VCP and its downstream components in existing yeast apoptosis network. VCP is important player in cancer cell survival and can be used as a target for cancer therapy. It also serves as detector of aggregated proteins that are known to be cause of neurodegenerative diseases such as Parkinson’s and Alzheimer’s. Our model showed that cell survival is mediated by VCP via the degradation of IkBα that leads to translocation of NFkB to nucleus. This result is in agreement with experimental findings that VCP is an essential target in the Akt signaling pathway suggesting that presented model in combination with experimental approach would represents a promising platform to study complex cellular processes involved in cancer and neurodegeneration.

Exploiting the advantages of BN models that enables extraction of system level properties of large networks and extensive state exploration and converting discrete model to continuous model our results suggested that in contrast to human apoptotic network yeast apoptosis is linear process whose regulation does not involve any complex feedback loops. Analysis of the second model showed that with certain adjustments Boolean model of yeast apoptosis can be adapted for studies of apoptosis in higher organisms.

Our results show that even without kinetic and qualitative data, it is possible to build models that can simulate relevant states of yeast physiology and regulations and can contribute to further understanding of biology. Since yeast is a preferred model organism for many studies of fundamental processes in a *Eukaryal* cell, we argue that *in silico* studies of yeast will be an important contributor to the understanding of complex cellular regulations, such as cell death pathways and that the applications will extend toward study of regulations or causes of human diseases such as cancer and neurodegeneration.

## Materials and Methods

### Apoptosis network setup

Based on the extensive literature study and databases search (*Saccharomyces* Genome Database-SGD; Cherry et al., 2011) the apoptosis network consisting of 73 nodes and 115 edges is constructed. These nodes are selected based on their interactions and their substrates involved in apoptosis. Cell Designer (Funahashi et al., 2003) was used to visualize genes and their connection. Cell Designer allowed us to represent network using comprehensive graphical notation. Moreover, Cell Designer is able to connect to online data bases such as KEGG (Kanehisa and Goto, 2000), BioModels (Li et al., 2010) and PubMed which expands its connectivity, visualization and model building. The most prominent advantage of Cell Designer is its ability to support SBML (Systems Biology Markup Language; Hucka et al., 2003) format. Creating SBML file avoids creating logic rule file which is more error prone when rules have to be defined by user in simple text and it is not in form of easily drawing boxes.

### Computation of logical steady states

Identification of Logical Steady States (LSSs) in a Boolean network is an important task as they comprise the states in which a gene-regulatory network resides most of the time. Strong biological implication can be carried out by LSSs of the network. LSSs can even be linked to phenotype (Kauffman, 1969). CNA (Klamt et al., 2006) is used to calculate all possible LLSs based on specified initial value for each gene and signal flow in network. LSSs are used to evaluate the network behavior under perturbation and changes in network structure.

### Identifying network wide dependency

**Figure 8:**
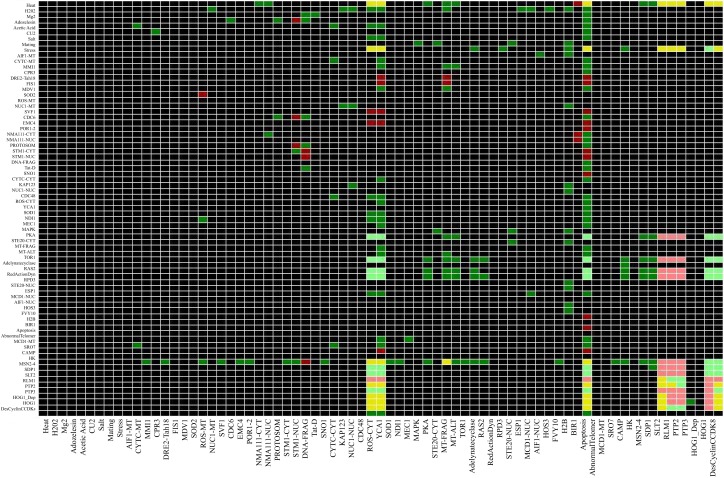
**Interaction Matrix of Yeast Apoptosis Network**. Each row corresponds to single species and each column corresponds to reactions. A red matrix element *e_ij_* indicates an inhibition influence of species *i*on reaction *j*. In contras a green filed shows an activation influence of species *i* on reaction j while a blue box indicates species *i* gets activated in reaction *j* and as it is expected the black cells indicates that species *i* does not participate in reaction *j*. Number of interactions where the species is involved is mentioned at the end of each row as connectivity number of each species. Number of reactions that activates/inhibits is mentioned in brackets (Color coding: red – inhibition, green – activation and black – no interaction influence).

**Figure 9:**
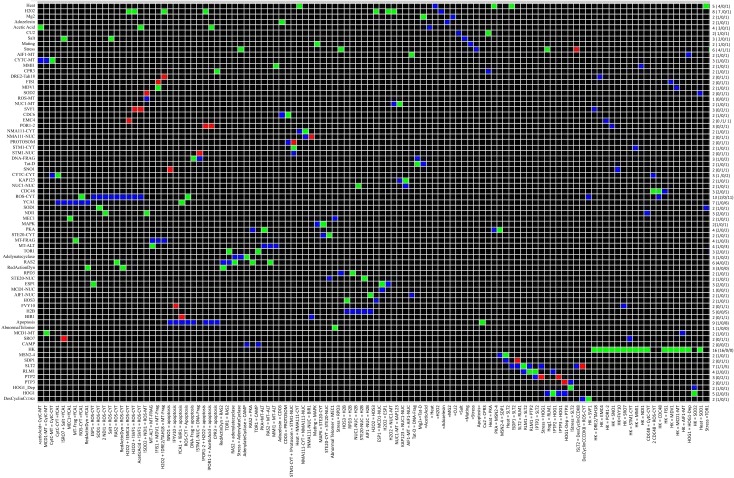
**Dependency Matrix for Yeast Apoptosis Network**. In the dependency matrix each element *m_ij_* represent the relation between an affecting and an affected species. Former is specified at the bottom of each column and later is shown at the beginning of each row. At intersection of *i*^th^ column and *j*^th^ row there are three possibilities. A yellow box indicates species *i*is an ambivalent factor meaning both activating and inhibiting path exist from species *i* to species *j*. Similarly a dark green and or light green cell shows a total and a non-total activator respectively. Another possibility is having dark or light red indicates species *i* is a total inhibitor or non-total inhibitor of species *j*. whenever there is no path from spices *i* to species *j* the intersection cell is field by black. The hyper graph underlying the network is a directed graph and consequently the dependency matrix is non-symmetric. (Color coding: light and dark green – complete and incomplete activation, dark and light red – complete and incomplete inhibitor, yellow indicates an ambivalent factor and black indicates that there is no dependency between two species).

Considering a pair of nodes *(a, b)* dependency is defined as the influence of node *a* on node *b* and vice versa, node *a* influences node *b* as follow: a is *a* total activator of *b* if there is an activating path between *a* and *b*, *a* is a total inhibitor of *b* is there is an inhibition path from *a* to *b* or a is an ambivalent effecter of b meaning that there is an intermediate node which is involved in a negative feedback loop. Using CNA the interaction matrix and dependency matrix were drawn from the network (Figures 8 and 9).

### Conversion of discrete to continuous model using SQUAD

In order to convert the schematic network into Boolean model we used SQUAD (Mendoza and Xenarios, 2006), a user friendly graphical software which is suitable for modeling signaling network where kinetic reactions are not available. Simulation in SQUAD consists of three steps: (1) the network is first loaded from the SBML file. Components of the network are presented as nodes and value of each node represents the state of that node. SQUAD converts the network to discrete dynamic model. Using Boolean algorithms all steady sates in network are calculated. (2) network is converted to a continues dynamic model generating sets of ordinary differential equations (ODEs) and steady states achieved in pervious step. (3) SQUAD allows perturbation in order to understand the role of each node within the network.

Using Reduced Order Binary Decision Diagram (ROBDD), it is possible to calculate steady states (Di Cara et al., 2007). ROBDD is a memory efficient data structure which is widely used in electronic field and has been proven to work for large binary networks. Moreover, ROBDD computes steady states for large networks (*n *> 50) in matter of seconds. Another advantage of this algorithm is its ability to identify the cyclic steady states. These oscillating states are reachable when system identifies a cyclic pattern instead of one single state.

In the first step, schematic network is supplied to SQUAD resulting in a discrete network, generating a set of either cyclic or single steady states. These steady states are then used to convert discrete model to continues. Given all calculated steady states or cycles we examined all possible outcomes for each input. Depending on the desire form of output, discrete to continuous conversion can be carried out either as a complete or progressive mode. Our simulations are performed in the complete mode since cell undergoing apoptosis should die after certain time and it is expected to maintain a constant level of apoptosis or survival (in case when apoptosis is not activated) at the end of each run. As an opposite to complete mode, the progressive model allows the user to stop the simulation at any time even before reaching the steady states.

### Conversion of discrete to continuous model using odefy

Reconstructing Boolean model of yeast apoptosis from qualitative knowledge never gives details about concentration of molecules in different time points. For this purpose the discrete Boolean model is transformed to continuous model using Odefy. Odefy uses the multivariate polynomial interpolation in order to transform the logical rules into sets of ODEs. Yeast apoptosis Boolean model is converted to continuous model using Hill Cube and normalized Hill Cube where the Hill function is normalized to the unit interval. Behavior of biochemical reactions can be seen as a sigmoid *Hill function* represented as $f\left(\bar{\bar{x}}\right)={\bar{\bar{x}}}^{n}∕\left({\bar{\bar{x}}}^{n}+k^{n}\right)$. Where, *n* is a Hill coefficient and determines the slope of the curve and is a measurement of cooperativity of the interactions, and parameter *k* corresponds to values 1 and 0 in the Boolean model in the following manner: threshold value above given *k* in Boolean model is considered as 1 (on state) and below as 0 (off state).

## Conflict of Interest Statement

The authors declare that the research was conducted in the absence of any commercial or financial relationships that could be construed as a potential conflict of interest.

## Research Topic:

From structural to molecular systems biology: experimental and computational approaches to unravel mechanisms of kinase activity regulation in cancer and neurodegeneration

## Topic Editor:

Matteo Barberis, Humboldt University Berlin, Germany; Max Planck Institute for Molecular Genetics, Berlin, Germany
